# First Nations Peoples in the forensic mental health system in New South Wales: Characteristics and rates of criminal charges post-release

**DOI:** 10.1177/00048674231151594

**Published:** 2023-02-14

**Authors:** Kimberlie Dean, Georgia Lyons, Anina Johnson, Elizabeth McEntyre

**Affiliations:** 1 Discipline of Psychiatry & Mental Health, School of Clinical Medicine, University of New South Wales, Sydney, NSW, Australia; 2 Justice Health and Forensic Mental Health Network, NSW, Australia; 3 Aboriginal-Led Research Consultant, Tea Gardens, NSW, Australia

**Keywords:** Forensic mental health, forensic patient, schizophrenia, First Nations People, reoffending

## Abstract

**Background:**

It is well established that First Nations Peoples in Australia are overrepresented within the criminal justice system. However, First Nations Peoples appear to be comparatively underrepresented in the forensic mental health system, and little is known about their outcomes once released from secure care.

**Objective:**

To compare the characteristics and rates of repeat criminal justice contact for a criminal charge of First Nations and non-First Nations forensic patients in New South Wales.

**Methods:**

Data on the sample were extracted from the New South Wales Mental Health Review Tribunal paper and electronic files matched to the Bureau of Crime Statistics and Research Reoffending Database. Characteristics of First Nations and non-First Nations patients were compared using univariate logistic regression analysis. Univariate and multivariate Cox proportional hazard regression was used to determine predictors of post-release criminal charges.

**Results:**

Key differences in the sociodemographic, clinical and forensic characteristics of First Nations compared with non-First Nations forensic patients were identified. The time to first criminal justice contact following release was significantly shorter for First Nations forensic patients (*p* < 0.01).

**Conclusion:**

The findings of this study confirm that First Nations forensic patients have distinct and complex needs that are apparent at entry to the forensic mental health system and that their poorer criminal justice contact rates following release from secure care indicate that these needs are not being adequately met either during treatment or once in the community. Responses to these study findings must consider the complex and continuing impact of colonisation on First Nations Peoples, as well as the need for solutions to be culturally safe.

## Introduction

Individuals affected by mental illnesses often face an elevated risk of contact with the criminal justice system (Fazel et al., 2009; Yee et al., 2020). Those with severe mental illnesses found to lack criminal responsibility for serious offences are often referred to as ‘forensic patients’. In many jurisdictions, including in Australia, the term ‘forensic patient’ refers particularly to those formally found ‘not criminally responsible or not guilty by reason of mental illness’ (NGMI) at court. While individuals with mental illness presenting to lower courts charged with less serious charges may also be diverted to health services for treatment (Soon et al., 2018), they are not typically referred to as ‘forensic patients’ and are not the focus of the present study. There is a substantial body of research focused on establishing outcomes for forensic patients following their release from secure mental health care, including rates of reoffending or repeat contact with the criminal justice system, maintenance of conditional release orders and patterns of health service contact. Reoffending rates among forensic patients are consistently found to be lower than for comparable groups, including released prisoners (Fazel et al., 2016). In a recent Australian study examining the rates and predictors of post-release justice contact among a cohort of 477 forensic patients, 6.3% of released NGMI forensic patients had a subsequent proven offence recorded over 12 months (Dean et al., 2020), much lower than local prison-release reconviction rates. Rates of reoffending may be higher among particularly groups of forensic patients, including those with a diagnosis of co-morbid personality disorder (Charette et al., 2015; Dean et al., 2020), co-morbid substance use problems (Simpson et al., 2018) and a prior criminal history (Green et al., 2011).

First Nations Peoples in Australia (i.e. Aboriginal and/or Torres Strait Islander People) are overrepresented within the criminal justice system, including in their contact with police, courts and prisons (Australian Institute of Health Welfare (AIHW), 2020; Baldry et al., 2015; Justice Health and Forensic Mental Health Network, 2015; McCausland et al., 2017). This overrepresentation must be understood in the context of the ongoing and intergenerational impacts of colonisation, dispossession, trauma and racism that First Nations Peoples in Australia experience (Australian Law Reform Commission [ALRC], 2018). Dispossession of land and waters, removal of children during the Stolen Generations period, the ongoing impact of statutory child protection systems, and the dismantling of cultural and kinship systems have all contributed to the high rates of criminal justice system contact, among other adverse outcomes. The inadequate cultural sensitivity, responsiveness and ongoing biases operating within existing systems and services are also likely to have further negative impacts for First Nations Peoples, with compounding effects on social and emotional wellbeing (Aboriginal Justice Victoria, 2022; Durey et al., 2014). The latest Network Patient Health Survey conducted by the New South Wales (NSW) Justice Health and Forensic Mental Health Network found that 66% of Aboriginal and/or Torres Strait Islander men and 80% of Aboriginal and/or Torres Strait Islander women in prison reported having received a diagnosis of mental illness (Justice Health and Forensic Mental Health Network, 2015). In addition to high rates of mental health problems and criminal justice contact, higher rates of repeated contact, including repeated incarceration, among First Nations Peoples have also been noted, with the Bureau of Crime Statistics and Research (BOCSAR) reporting that in 2017, 52.1% of released prisoners with an Aboriginal and/or Torres Strait Islander background had repeated contact with the justice system within 12 months post-release (New South Wales Bureau of Crime Statistics and Research (NSW BOCSAR), 2020).

Although diversion into mental health treatment services is a key strategy to address the high rates of mental illness among people in contact with the criminal justice system, Aboriginal and/or Torres Strait Islander People may face particular barriers to accessing mental health diversion (Soon et al., 2018). First Nations Peoples also appear to be comparatively underrepresented in the forensic mental health system, when compared with their overrepresentation in the criminal justice system. In 2016, the NSW Justice Health and Forensic Mental Health Network estimated that 16.8% of the forensic patient population in high secure settings were of Aboriginal and/or Torres Strait Island background (Justice Health and Forensic Mental Health Network, 2016), compared with the rate of 29% in the Australian prison population (Australian Bureau of Statistics [ABS], 2020). It is important to consider biases which may be operating in the legal, justice and forensic mental health systems, including whether a mental health defence is less often considered or granted for Indigenous Australians (Ellis et al., 2010). First Nations Peoples also face challenges in obtaining an accurate mental health diagnoses, as some disorders may be misdiagnosed or underdiagnosed (MacGillivray and Baldry, 2013).

Despite the clear need for evidence to inform the development of culturally appropriate responses to First Nations Peoples within or trying to access the forensic mental health system, there has been limited research undertaken in this field to date. One Australian study of 364 NGMI forensic patients (a subset of patients from the present study population) found that individuals who identified as Aboriginal and/or Torres Strait Islander were more likely to have a post-release offence recorded compared with those from a non-Aboriginal background (Hayes et al., 2014). There is also some emerging evidence to suggest that First Nations Peoples within the forensic mental health system may continue to experience adversity, with one survey of patients in high secure care identifying high rates of self-reported ongoing racial discrimination (Justice Health and Forensic Mental Health Network, 2016).

The current study aims to address gaps in our understanding of the profiles and outcomes for First Nations forensic patients by comparing sociodemographic, clinical and forensic characteristics with those of non-First Nations forensic patients and by examining the comparative rates and predictors of post-release criminal justice contact in the form of recorded criminal charges.

## Method

### First Nations Community and cultural governance arrangements

The research was initiated and co-led by Aboriginal-Led Research Consultant E.M., along with K.D., academic lead for the NSW Forensic Patient Database project. From its initial planning stages the research was designed and developed in collaboration with members of NSW Aboriginal Communities. Worimi and Wonnarua Woman and Elder Dr Elizabeth McEntyre, recognised as a leading Aboriginal researcher and professional in criminal justice, disability and mental health systems in NSW, engaged with Aboriginal Community Controlled Health Services, Local Aboriginal Land Councils and Aboriginal-led groups with regard to the intent and potential value of the proposed research to First Nations Peoples and Communities. Mindaribba Local Aboriginal Land Council (located on Wonnarua Country) and Biripi Aboriginal Corporation Medical Centre (located on Biripi Country), whose deliberate efforts are aimed at ensuring that First Nations Peoples can live better lives, provided formal support for the research, with the latter having ongoing oversight. E.M. had regular discussions with both Aboriginal-Led organisations and with community members, in order to safeguard all aspects of the study, including the generation of the original research questions, the study design and analysis, the interpretation of results and the reporting/publication of findings. The project was Aboriginal co-led, Aboriginal co-produced and Aboriginal-informed from beginning to end. Overall, the proposed research, and indeed all Aboriginal-led research which has a focus on criminal justice, disability and mental health systems, were pursued on the basis that they reflected the critical interests and unrelenting concerns of Aboriginal People and Communities in NSW, highlighted by their lived experience and reality, along with community crime records and correctional statistics, which confirms the near-ubiquitous experience of justice contact for Aboriginal families and Communities (McEntyre, 2019). The current paper details findings from the quantitative component of a wider programme of research focused on First Nations Peoples’ experiences and outcomes in relation to the forensic mental health system in NSW. All requirements of the NSW Aboriginal Health and Medical Research Council of NSW Human Research Ethics Committee were also met (HREC Reference: 1749/20).

### Sampling and data collection

Under the *Mental Health (Forensic Provisions) Act 1990* (NSW), the NSW Mental Health Review Tribunal (MHRT) was responsible for making orders regarding the care, treatment and supervision of forensic patients.^1^ Data for the current study were extracted from MHRT electronic records and paper case files for individuals receiving an NGMI verdict between 1 January 1990 and 29 July 2016. Data extracted from paper case files included sociodemographic variables; including whether patients had a First Nations background, as well as a range of clinical and criminal justice information. Specifically, the paper case files included relevant legal documents, such as police fact sheets detailing offences, criminal records, judicial decisions and MHRT decisions, and progress reports prepared by treating clinicians for submission to the MHRT. Diagnostic information extracted from the paper case files was not necessarily based on structured diagnostic interviews, but rather was based on the clinician’s recorded diagnosis in the files (Dean et al., 2020).

The MHRT electronic database also identified whether patients had been granted conditional or unconditional release from secure care during the data collection period (up until 30 November 2017). Conditional release allows a forensic patient to be released from detention on the basis that they will adhere to specific conditions, such as abstaining from drugs and/or alcohol, living in specified accommodation, attending appointments with mental health professionals and complying with prescribed medication. Unconditional release allows a forensic patient to be released from formal supervision by the MHRT. Breach of conditional release does not automatically result in criminal justice system contact or consequence (i.e. unlike parole or probation orders). Supervision and monitoring of forensic patients under conditional release is undertaken by health and not justice staff.

The sample was then linked to NSW state-wide criminal justice datasets to examine patients’ criminal justice outcomes following release from secure care. This included the BOCSAR Reoffending Database (ROD), which contains information related to criminal charges, convictions and mental health disposals from 1994 until 30 November 2017. The ROD contains details on the type of offence, date of offence, charge outcome and penalties imposed for all finalised charges recorded in NSW. Data from the ROD were obtained using a record linkage process, where identifying information for cohort members (i.e. names, aliases, dates of birth, sex, study IDs, NSW Department of Corrections Master Index Numbers, and Department of Health Medical Record Number) were provided by the NSW MHRT to BOCSAR to enable matching of cohort members to information contained in the ROD (Dean et al., 2020). Any individuals who could not be linked to the ROD were excluded from any analysis of the rate of criminal charges post-release.

### Data analysis

Descriptive statistics were generated to establish the sociodemographic, clinical and criminal justice characteristics of the cohort. The First Nations and non-First Nations cohorts were compared on these key variables using univariate logistic regression analysis. Data relating to charges, court outcomes and offence type, categorised according to the Australian and New Zealand Society of Criminology (ANZSOC) classification system (ABS, 2011), were analysed to determine the prevalence and nature of post-release criminal charges.

Kaplan–Meier survival analysis was used to determine the time to first post-release charge, stratified by First Nations status. Univariate and multivariate Cox proportional hazard regression was used to determine predictors of post-release charges. The significant univariate predictors for post-release charges (any offence type and violence offence) identified by Dean et al. (2020) were included in the multivariate model using a step-wise approach, removing any non-significant variables at each stage of testing (see Supplementary Table 1 for the univariate analysis). Cell sizes of less than five were not reported in order to minimise the risk of re-identification. All statistical analyses were performed using Statistical Package for the Social Sciences (SPSS) version 26.

### Ethical review

Ethical approval for this research was provided by the Population and Health Services Research Ethics Committee (AU RED Reference: HREC/18/CIPHS/48; Cancer Institute NSW Reference: 2018HRE1003) and the Aboriginal Health and Medical Research Council of NSW (HREC Reference: 1749/20).

## Results

### Sample characteristics

Of the 477 forensic patients (found NGMI) in the study sample, 11.3% (*n* = 54) were identified as having a First Nations background. Table 1 compares key sociodemographic, clinical and forensic characteristics for the First Nations and non-First Nations patients in the sample. First Nations patients were significantly more likely to be female (OR = 2.30, 95% CI = [1.16, 4.58]) and from an English-speaking background (OR = 7.59, 95% CI = [2.32, 24.80]). At the time of the index offence, they were also more likely to have children (OR = 3.45, 95% CI = [1.88, 6.35]), to have not completed year 10 (OR = 3.70, 95% CI = [2.05, 6.68]) and not to have been engaged in employment or study (OR = 5.30, 95% CI = [1.61, 17.44]). First Nations forensic patients were also significantly more likely to have a recorded history of child abuse or neglect (OR = 2.27, 95% CI = [1.22, 4.23]).

**Table 1.** Sample characteristics and differences between First Nations and non-First Nations forensic patients.

		First Nations background (*n* = 54)	Non-First Nations background (*n* = 423)	OR [95% CI]	*p*-value
		*n* (%)	*n* (%)		
Sociodemographic characteristics					
Sex	Male	41 (75.9)	370 (87.5)	1.00 (reference)	**0.018**
	Female	13 (24.1)	51 (12.1)	2.30 [1.16, 4.58]	
Language background	Non-English background	<5	128 (30.3)	1.00 (reference)	**0.001**
	English background	50 (92.6)	281 (66.4)	7.59 [2.32, 24.80]	
Marital status	Single/never married	21 (38.9)	197 (46.6)	1.00 (reference)	0.469
	Other	18 (33.3)	132 (31.2)	1.28 [0.66, 2.49]	
Parental status	No children	17 (31.5)	261 (61.7)	1.00 (reference)	**<0.001**
	Has children	36 (66.7)	160 (37.8)	3.45 [1.88, 6.35]	
Education level	Completed year 10	23 (42.6)	299 (70.7)	1.00 (reference)	**<0.001**
	Below year 10	29 (53.7)	102 (24.1)	3.70 [2.05, 6.68]	
Any employment/study	Yes	<5	100 (23.6)	1.00 (reference)	**0.006**
	No	45 (83.3)	283 (66.9)	5.30 [1.61, 17.44]	
History of child abuse/neglect	No	17 (31.5)	206 (48.7)	1.00 (reference)	**0.010**
	Yes	32 (59.3)	171 (40.4)	2.27 [1.22, 4.23]	
Clinical characteristics					
Most recent diagnosis	Other	8 (14.8)	67 (15.8)	1.00 (reference)	0.835
	Schizophrenia-related	46 (85.2)	354 (83.7)	1.09 [0.49, 2.41]	
Personality disorder diagnosis	No	36 (66.7)	353 (83.5)	1.00 (reference)	**0.003**
	Yes	15 (27.8)	54 (12.8)	2.72 [1.40, 5.31]	
Psychopathy diagnosis	No	45 (83.3)	381 (90.1)	1.00 (reference)	0.272
	Yes	5 (9.3)	24 (5.7)	1.76 [0.64, 4.85]	
Substance use disorder diagnosis	No	<5	160 (37.8)	1.00 (reference)	**<** **0.001**
	Yes	49 (90.7)	261 (61.7)	10.01 [3.07, 32.66]	
Intellectual disability	No	20 (37.0)	225 (53.2)	1.00 (reference)	**0.002**
	Yes	11 (20.4)	35 (8.3)	3.54 (1.56, 8.01)	
History of head injury	No	21 (38.9)	250 (59.1)	1.00 (reference)	**0.002**
	Yes	28 (51.9)	131 (31.0)	2.55 [1.39, 4.66]	
History of self-harm	No	10 (18.5)	160 (37.8)	1.00 (reference)	**0.004**
	Yes	42 (77.8)	234 (55.3)	2.87 [1.40, 5.89]	
First degree relative with mental illness	No	15 (27.8)	187 (44.2)	1.00 (reference)	**0.006**
	Yes	31 (57.4)	156 (36.9)	2.48 [1.29, 4.76]	
Prior mental health contact	No	10 (18.5)	79 (18.7)	1.00 (reference)	0.971
	Yes	44 (81.5)	343 (81.1)	1.01 [0.49, 2.10]	
Forensic characteristics					
Index offence	Homicide and related act	21 (38.9)	225 (53.2)	1.00 (reference)	**0.048**
	Other	33 (61.1)	197 (46.6)	1.80 [1.01, 3.20]	
Prior charge	No	<5	177 (41.8)	1.00 (reference)	**<0.001**
	Yes	49 (90.4)	227 (53.7)	9.55 [3.38, 26.97]	
Prior violent charge	No	14 (25.9)	244 (57.7)	1.00 (reference)	**<0.001**
	Yes	39 (72.2)	160 (37.8)	4.25 [2.24, 8.08]	
Prior imprisonment	No	28 (51.9)	347 (82.0)	1.00 (reference)	**<0.001**
	Yes	25 (46.3)	57 (13.5)	5.44 [2.96, 9.98]	

CI: confidence interval; OR: odds ratio.

Cell sizes less than 5 have not been reported to minimise the risk of re-identification. Significant results are shown in bold.

First Nations forensic patients were more likely than non-First Nations patients to have a diagnosis of co-morbid personality disorder (OR = 2.72, 95% CI = [1.40, 5.31]), co-morbid substance use disorder (OR = 10.01; 95% CI = [3.07, 32.66]) and intellectual disability (OR = 3.54, 95% CI = [1.56, 8.01]). They were also more likely to have a history of head injury (OR = 2.55, 95% CI = [1.39, 4.66]), self-harm (OR = 2.87, 95% CI = [1.40, 5.89]) and to have a first-degree relative with a mental illness (OR = 2.48, 95% CI = [1.29, 4.76]). They were more likely to have a non-homicide-related index offence leading to the NGMI finding (OR = 1.80, 95% CI = [1.01, 3.20]) and were more likely to have a history of prior criminal charges (OR = 9.55, 95% CI = [3.38, 26.97]), as well as a prior term of imprisonment (OR = 5.44, 95% CI = [2.96, 9.98]).

### Post-release criminal charges

Of the 477 forensic patients in the sample, 96.2% (*n* = 459) were successfully matched to the BOCSAR ROD and could be included in the analysis. Of these patients, 282 had been conditionally or unconditionally released before 30 November 2017 (28 First Nations patients; 254 non-First Nations patients). Among the First Nations patients, 39.3% (*n* = 11) had at least one charge recorded as occurring post-release (either conditional or unconditional release; whichever came first), compared with 19.7% (*n* = 50) for the non-First Nations sample.

**Figure 1.**
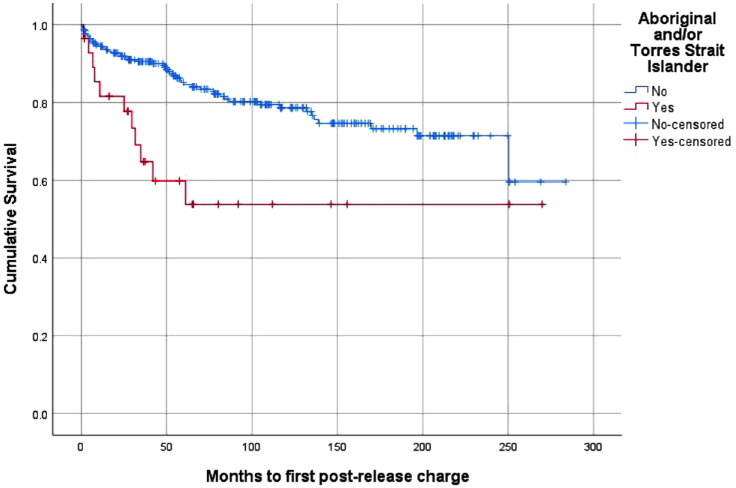
Survival curve – post-release charges, stratified by First Nations status.

Figure 1 shows the survival curves for post-release charges for any offence, stratified by First Nations background. The mean survival time to first post-release charge for any offence was 214.40 months (95% CI = [199.11, 229.70]) for the sample overall – 157.24 months (95% CI = [106.86, 207.62]) for the First Nations cohort and 218.38 months (95% CI = [203.24, 235.51]) for the non-First Nations cohort. The difference in survival time between the two groups was statistically significant (*p* < 0.01).

Table 2 presents the results of univariate Cox proportional hazards regression for any criminal charges recorded as occurring post-release for the sample as a whole. First Nations background was found to be significantly associated with having a post-release charge for any reason (HR = 2.68; 95% CI = [1.39, 5.20]). Multivariate analysis was also used to establish factors independently associated with receiving a post-release charge. The results from step-wise multivariate analysis are presented in Table 3. This was conducted by including those variables previously identified as being significantly associated with repeat criminal justice contact on univariate analysis and detailed in a previous publication by Dean et al. (2020) (see Supplementary Table 1), which was performed using the same sample as the current study. After adjusting for all significant covariates, having a First Nations background (HR = 2.19; 95% CI = [1.06, 4.52]) and having a co-morbid diagnosis of personality disorder (HR = 2.51; 95% CI = [1.25, 5.04]) both remained significantly associated with post-release charges for any offence.

**Table 2.** Univariate Cox proportional hazards regression analysis examining association between First Nations background and incidence of any post-release charge.

		Total *N*	Any charge *n* (%)	Incidence rate (per 100-person years)	HR [95% CI]	*p*-value
Post-release charge						
First Nations background	No	254	50 (19.7)	2.41	1.00 (reference)	**0.003**
	Yes	28	11 (39.3)	7.59	2.69 [1.39, 5.20]	

CI: confidence interval; HR: hazard ratio.

Significant results are shown in bold.

**Table 3.** Multivariate Cox proportional hazards regression analysis examining associations between First Nations background and incidence of any post-release charge, along with key covariates.

Covariates	Model 1 HR [95% CI]	Model 2 HR [95% CI]	Model 3 HR [95% CI]
First Nations background	**2.46 [1.26, 4.78];***p* **=** **0.008**	**2.40 [1.21, 4.79];***p* **=** **0.013**	**2.19 [1.06, 4.52];***p* **=** **0.035**
English-speaking background	1.71 [0.92, 3.19]; *p* = 0.092		
Personality disorder diagnosis		**2.50 [1.29, 4.85];***p* **=** **0.006**	**2.51 [1.25, 5.04];***p* **=** **0.010)**
Substance use disorder diagnosis		**2.00 [1.06, 3.78];***p* **=** **0.034**	1.74 [0.86, 3.56]; *p* = 0.126
Charge prior to the index offence			1.75 [0.92, 3.33]; *p* = 0.091

CI: confidence interval; HR: hazard ratio.

Significant results are shown in bold.

## Discussion

This study addresses a significant gap in the evidence base regarding the profile and criminal justice outcomes for First Nations Peoples in contact with the forensic mental health system in Australia. The study findings identified significant challenges and hardships for First Nations forensic patients experienced prior to entering the forensic mental health system, consistent with the known impacts of long-standing and systemic disadvantage and adversity dating back to colonisation. The findings also confirm an apparent underrepresentation of First Nations Peoples in the forensic mental health system, given the hyper-incarceration rates for First Nations people. Finally, First Nations forensic patients were found to have higher rates of post-release criminal charges compared with non-First Nations forensic patients, raising the possibility that the needs of First Nations forensic patients are not being adequately addressed by the forensic mental health and other systems. Overall, the study findings indicate that First Nations forensic patients have complex wellbeing and support needs both at entry to the forensic mental health system and on release, necessitating consideration of the development and implementation of culturally appropriate responses informed by the known historical physical, social, financial and cultural determinants of health and wellbeing for First Nations communities.

### Main findings

When compared with non-First Nations forensic patients, First Nations forensic patients were significantly more likely to enter the forensic mental health system with a range of indicators of sociodemographic disadvantage and were more often diagnosed with other mental health problems in addition to a primary psychotic illness (including co-morbid personality disorder, co-morbid substance use disorder and intellectual disability and a history of head injury or of self-harm). While the high levels of sociodemographic disadvantage for First Nations Peoples in Australia are well established and understood in terms of the impact of colonisation, dispossession, trauma, racism and ongoing systemic discrimination, the current study further confirms the importance of these factors and this context specifically for First Nations people entering the forensic mental health system. Similarly, given the overrepresentation of First Nations Peoples within the criminal justice system that arises from the same context, it was unsurprising that First Nations forensic patients were also more likely to have a history of contact with the criminal justice system prior to entering the forensic mental health system. Given that these indicators are likely to be associated with poorer long-term outcomes, culturally informed and targeted interventions addressing the underlying causes of repeated criminal justice contact are needed, with implementation required at a systemic and not only individual level.

An important finding of the present study relates to the apparent underrepresentation of First Nations Peoples in the forensic mental health system in light of elevated incarceration rates in the general prison population. While almost one-third of the Australian prison population identifies as Aboriginal and/or Torres Strait Islander (ABS, 2020), they comprised only 11.3% of the cohort in the current study. It is important to consider why First Nations Peoples appear to be less likely to receive a mental health defence and be diverted from the criminal justice system to forensic mental health services. It may be that First Nations Peoples are less likely to have their mental health problems identified, are less inclined to seek support, and are not fully informed about the forensic mental health system or their mental health defence options at court. Negative perceptions may also be held by First Nations Peoples, their families, and advising professionals regarding mental illness, the implications of making a mental health defence at court and about the forensic mental health system itself. These possibilities require further investigation if the barriers to access to forensic mental health services are to be addressed. Existing research also highlights that First Nations People may be less likely to receive a mental health diversion when presenting to the lower courts, even if deemed to be eligible by mental health clinicians (Soon et al., 2018), and it is likely some of the same biases and barriers may be operating in these two legal contexts. Finally, First Nations Peoples with mental health problems may be less likely to be granted a mental health defence when it is submitted or may be more likely to be charged with lesser offences for which the defence is not appropriate. Further research, particularly qualitative research, is needed to better understand the factors contributing to this apparent underrepresentation.

Consistent with findings for forensic patients across jurisdictions internationally, First Nations forensic patients released from secure care in the current study had lower rates of post-release criminal charges compared with those typically reported for prison-release cohorts, providing some support for the positive impact of treatment and supervision in the forensic mental health system. However, First Nations forensic patients had substantially higher rates of post-release charges compared with non-First Nations forensic patients. By the end of the follow-up period, almost 40% of the First Nations patients had a criminal charge recorded, compared with almost 20% of non-First Nations patients, with the time to first charge post-release being significantly different. This difference calls into question the effectiveness of existing systems, services and interventions in adequately meeting the needs and risks of First Nations forensic patients who have been released to live in the community. The extent to which the increased rate of post-release charges for First Nations forensic patients is due to biases in the criminal justice system is also important to consider, although the data available in the current study did not allow for any direct examination of this possibility. The high rates of indicators of systemically generated adversity that were evident prior to entry into the forensic mental health system for First Nations forensic patients may represent risk factors for future post-release outcomes and may need to be more comprehensively assessed and addressed prior to release.

While there are no published studies of culturally led or informed interventions for First Nations forensic patients in Australia, the findings of the current study strongly support the need for such approaches to be developed and tested. In the broader criminal justice system context in Australia, similar calls have been made in relation to health and social support programmes for First Nations people transitioning from prison to the community (Abbott et al., 2018; Kendall et al., 2020). Access to First Nations controlled community-based health services for those receiving treatment in secure settings is one approach that should be considered, given the success demonstrated in a custodial context (Arthur et al., 2022). It is also clear that professionals working with forensic patients may feel unable to provide adequate care for First Nations patients, with a survey of forensic mental health professionals in Western Australia (Durey et al, 2014) identifying a range of service-related factors that staff reported had compromised their ability to deliver high-quality and culturally safe care, including a shortage of resources to support the wellbeing of Aboriginal patients and the need to have better links with Aboriginal-led health and support services. Beyond the Australian context, Indigenous models of care for First Nations Peoples in prison settings have been proposed and developed in several international jurisdictions (Perdacher et al., 2019).

### Strengths and limitations

The current study benefits from several strengths, including the relatively large total-population sample of forensic patients in contact with forensic mental health services in NSW over a 25-year period. Outcome data were obtained from official criminal justice records using a record-linkage methodology, which limits the potential for sampling and information biases. However, the limitations of the study must also be noted. The quality of data obtained from the MHRT varied, with some variables subject to missing data. Data obtained from the BOCSAR ROD were limited to charges recorded between 1994 and November 2017, meaning that any charges recorded outside this date range could not be included. Offending behaviour which did not result in criminal charges was also unrecorded in the available data. Some data analysis may have been underpowered given the smaller sample of First Nations forensic patients who had received conditional or unconditional release. Data were not available to examine a number of factors likely to be important for explaining associations found, including indicators of cultural identity, connection to community and other sources of strength and resilience for First Nations Peoples. Similarly, it was not possible to directly examine systemic biases or other underlying causes of the poorer outcomes found for First Nations forensic patients. The findings of this study may not be generalisable to other jurisdictions given the varying legal and forensic mental health service approaches to forensic patients nationally.

## Conclusion

This study has addressed a significant gap in the evidence base by focusing on First Nations Peoples in contact with the forensic mental health system and is the first to examine profiles and patterns of criminal justice contact after forensic mental health care. While the findings of this study suggest that the treatment and support provided to First Nations forensic patients released to live in the community may be effective to some extent in preventing post-release criminal justice contact, the contrast in outcomes compared with non-First Nations patients highlights that significant needs remain unmet and systemic sources of bias likely remain unaddressed for this group.

Current approaches to the care and treatment of forensic patients in Australia may be inadequate for meeting the needs of First Nations Peoples. More also needs to be done to understand the apparent underrepresentation of First Nations People in the forensic mental health system to ensure any discriminatory barriers to access to care are removed. It is critically important to recognise the unique needs, including cultural and community-connection needs, of First Nations forensic patients, who are also more likely to begin their forensic mental healthcare journey with a significant history of complex trauma and considerable hardship arising from systemic failures and neglect. A more proactive approach is necessary, one that begins with the early identification of needs and the engagement of First Nations People in all aspects of the development, delivery and evaluation of models of mental healthcare, in order to prevent those involved in the forensic mental health system from having further contact with the criminal justice system following release back into the community. Having access to ongoing Aboriginal-led wrap-around services, including community-based services controlled by First Nations people, for as long as required to address complex support needs is essential. Histories of generations of existence, resistance and personal and collective trauma for First Nations Peoples should be considered in the redesign and delivery of all forensic mental health programmes and services to ensure all practice is person and collectively centred and culturally responsive.

## Supplemental Material

sj-docx-1-anp-10.1177_00048674231151594 – Supplemental material for First Nations Peoples in the forensic mental health system in New South Wales: Characteristics and rates of criminal charges post-release Click here for additional data file. Supplemental material, sj-docx-1-anp-10.1177_00048674231151594 for First Nations Peoples in the forensic mental health system in New South Wales: Characteristics and rates of criminal charges post-release by Kimberlie Dean, Georgia Lyons, Anina Johnson and Elizabeth McEntyre in Australian & New Zealand Journal of Psychiatry

## Notes

1. It should be noted that in 2021 the *Mental Health (Forensic Provisions) Act 1990* (NSW) was replaced with the *Mental Health and Cognitive Impairment Forensic Provisions Act 2020* (NSW). The legislation replaced the term ‘NGMI’ (not guilty by reason of mental illness) with a finding of ‘act proven but not criminally responsible’. However, the term ‘NGMI’ has been retained throughout this paper as all data were collected under the previous legislation.
