# Supplementary material for: First Nations Peoples in the forensic mental health system in New South Wales: Characteristics and rates of criminal charges post-release
Source: Aust N Z J Psychiatry. 2023 Feb 14;57(6):904–13. doi: 10.1177/00048674231151594 (PMC10201079; doi:10.1177/00048674231151594)
Supplement: sj-docx-1-anp-10.1177_00048674231151594 – Supplemental material for First Nations Peoples in the forensic mental health system in New South Wales: Characteristics and rates of criminal charges post-release [file sj-docx-1-anp-10.1177_00048674231151594.docx]

**Supplementary Table 1.** Univariate Cox proportional hazards regression analysis of incidence of post-release criminal charges (as published in Dean et al. 2020).

|  | | Total N | Any charge  n (%) | Incidence rate (per 100-person years) | Hazard Ratio (95% CI) | p-value |
| --- | --- | --- | --- | --- | --- | --- |
| *Sociodemographic characteristics* | | | | |  | |
| Sex | Female | 47 | 9 (19.1) | 2.77 | 1.00 (reference) | 0.942 |
|  | Male | 234 | 52 (22.2) | 2.74 | 1.03 (0.51-2.09) |  |
| Language background | Non-English speaking | 78 | 13 (16.7) | 1.62 | 1.00 (reference) | **0.047** |
|  | English speaking | 198 | 48 (24.2) | 3.46 | 1.87 (1.01-3.46) |  |
| Relationship status | Other | 94 | 22 (23.4) | 2.68 | 1.00 (reference) | 0.899 |
|  | Single/never married | 138 | 34 (24.6) | 2.75 | 1.04 (0.61-1.77) |  |
| Parental status | No children | 167 | 31 (18.6) | 2.36 | 1.00 (reference) | 0.162 |
|  | Has children | 112 | 30 (26.8) | 3.38 | 1.43 (0.87-2.37) |  |
| Education level | Below year 10 | 71 | 14 (19.7) | 2.52 | 1.00 (reference) | 0.582 |
|  | Completed year 10 | 196 | 45 (23.0) | 2.88 | 1.18 (0.65-2.16) |  |
| Employment or study | Yes | 71 | 13 (18.3) | 2.07 | 1.00 (reference) | 0.271 |
|  | No | 183 | 44 (24.0) | 3.01 | 1.42 (0.76-2.63) |  |
| History of child abuse or neglect | No | 144 | 29 (20.1) | 2.26 | 1.00 (reference) | 0.058 |
|  | Yes | 107 | 26 (24.3) | 4.50 | 1.68 (0.98-2.86) |  |
| *Clinical characteristics* | | | | | | |
| Primary diagnosis | Other | 56 | 12 (21.4) | 2.57 | 1.00 (reference) | 0.827 |
|  | Schizophrenia-related | 226 | 49 (21.7) | 2.79 | 1.07 (0.57-2.02) |  |
| Co-morbid personality disorder | No | 239 | 45 (18.8) | 2.29 | 1.00 (reference) | **0.001** |
|  | Yes | 31 | 12 (38.7) | 8.33 | 3.03 (1.59-5.77) |  |
| Substance use disorder diagnosis | No | 97 | 13 (13.4) | 1.33 | 1.00 (reference) | **0.003** |
|  | Yes | 183 | 47 (25.7) | 3.88 | 2.58 (1.39-4.80) |  |
| Intellectual disability | No | 166 | 40 (24.1) | 2.80 | 1.00 (reference) | 0.095 |
|  | Yes | 27 | 9 (33.3) | 5.64 | 1.86 (0.90-3.85) |  |
| History of head injury | No | 179 | 33 (18.4) | 2.24 | 1.00 (reference) | 0.059 |
|  | Yes | 79 | 21 (26.6) | 4.00 | 1.70 (0.98-2.94) |  |
| History of self-harm | No | 110 | 19 (17.3) | 1.95 | 1.00 (reference) | 0.061 |
|  | Yes | 157 | 41 (26.1) | 3.50 | 1.68 (0.98-2.90) |  |
| First degree relative with mental illness | Yes | 100 | 16 (16.0) | 2.14 | 1.00 (reference) | 0.077 |
|  | No | 125 | 33 (26.4) | 3.66 | 1.72 (0.94-3.12) |  |
| Prior mental health contact | No | 53 | 11 (20.8) | 2.14 | 1.00 (reference) | 0.446 |
|  | Yes | 229 | 50 (21.8) | 2.93 | 1.29 (0.67-2.48) |  |
| *Forensic characteristics* | | | | | | |
| Index offence | Homicide-related | 198 | 40 (20.2) | 2.54 | 1.00 (reference) | 0.407 |
|  | Other | 84 | 21 (25.0) | 3.23 | 1.25 (0.73-2.12) |  |
| Prior charge | No | 142 | 25 (17.6) | 1.74 | 1.00 (reference) | **0.003** |
|  | Yes | 140 | 36 (25.7) | 4.59 | 2.22 (1.32-3.75) |  |
| Prior violent charge | No | 193 | 37 (19.2) | 2.08 | 1.00 (reference) | **0.004** |
|  | Yes | 89 | 24 (27.0) | 5.38 | 2.17 (1.28-3.67) |  |
| Prior imprisonment | No | 256 | 52 (20.3) | 2.48 | 1.00 (reference) | **0.014** |
|  | Yes | 26 | 9 (34.6) | 7.07 | 2.44 (1.20-4.99) |  |

CI: Confidence Interval.

Significant results are shown in bold.
